# Martial arts training as a psychological self-regulation intervention: an experimental study on emotional control, attention, and stress resilience

**DOI:** 10.3389/fpsyg.2026.1787259

**Published:** 2026-03-02

**Authors:** Cheng Zheng, Jiabing Zhou, Canzhong Ji

**Affiliations:** 1 School of Sports Science and Physical Education, Nanjing Normal University, Nanjing, Jiangsu, China; 2 School of Physical Education, Guangdong University of Petrochemical Technology, Maoming, Guangdong, China

**Keywords:** attention, emotion regulation, martial arts, resilience, self-regulation, stress

## Abstract

**Background:**

Psychological self-regulation, encompassing emotion regulation, attentional control, and stress resilience, is a critical determinant of mental health and adaptive functioning. While conventional physical exercise has demonstrated psychological benefits, emerging evidence suggests that martial arts training may provide unique advantages due to its integration of physical exertion, cognitive engagement, and emotional regulation. However, randomized controlled trials directly comparing martial arts training with conventional exercise using multimodal outcome measures remain limited.

**Methods:**

Sixty-six healthy young adults (18–25 years) were randomly assigned to a martial arts self-regulation training group (MA-SRT), a conventional physical exercise group (CPEG), or a passive control group (*n* = 22 per group). The intervention lasted 8 weeks, with three 60 min sessions per week. Primary outcomes included emotion regulation (ERQ), attentional control (continuous performance task reaction time), and perceived stress (PSS-10). Secondary outcomes assessed psychological resilience (CD-RISC-25), electro-dermal activity, and executive inhibitory control (Stroop task). Outcomes were measured pre- and post-intervention. Data were analyzed using mixed-model repeated-measures ANOVA with effect sizes reported as partial eta squared and Cohen’s *d*.

**Results:**

Significant group × time interactions were observed for all primary and secondary outcomes (all *p* < 0.001). The MA-SRT group demonstrated the largest improvements in emotion regulation, attentional performance, and stress reduction, with large effect sizes (*η*^2^*_p_* = 0.49–0.75), whereas the CPEG showed moderate improvements and the control group minimal change. Secondary outcomes similarly favored the MA-SRT group, including marked gains in psychological resilience, reductions in sympathetic arousal, and enhanced executive control (*η*^2^*_p_* = 0.53–0.71).

**Discussion:**

These findings indicate that martial arts training is a highly effective psychological self-regulation intervention, producing superior emotional, cognitive, and physiological benefits compared to conventional physical exercise. The integrative cognitive-emotional demands inherent in martial arts practice may uniquely enhance self-regulatory capacity in young adults.

## 1 Introduction

Psychological self-regulation, the capacity to modulate cognition, emotion, and behavior in accordance with contextual demands, represents a fundamental determinant of adaptive functioning and mental health (Gross, 2015; Baumeister and Vohs, 2004). Contemporary research has increasingly recognized the role of physical activity in enhancing self-regulatory capacity (Denson et al., 2014), yet questions remain regarding which forms of exercise optimally facilitate psychological self-regulation. While conventional physical exercise interventions have demonstrated benefits for stress reduction and cognitive functioning (Salmon, 2001; Hillman et al., 2008), emerging evidence suggests that martial arts training may offer unique advantages due to its integration of physical, cognitive, and contemplative elements (Lakes and Hoyt, 2004; Vertonghen and Theeboom, 2010).

Martial arts practice encompasses structured physical movements combined with explicit demands for attentional focus, emotional control, and mindfulness-based awareness. Unlike conventional exercise modalities that primarily target physiological fitness, martial arts training requires practitioners to maintain sustained attention during complex movement sequences, regulate emotional responses during controlled combat scenarios, and cultivate present-moment awareness through coordinated breathing and movement (Woodward, 2009). Research has shown that martial arts practitioners demonstrate superior executive functioning and emotional regulation compared to non-practitioners (Diamond and Ling, 2016; Moore and Malinowski, 2009), suggesting that the cognitive-emotional demands embedded within martial arts training may facilitate broader self-regulatory competencies. However, the majority of existing studies have relied on cross-sectional designs comparing experienced martial artists with control populations, limiting causal inference regarding the specific effects of martial arts training on psychological self-regulation (Bu et al., 2010; Douris et al., 2015).

The theoretical framework for understanding martial arts effects on self-regulation draws upon multiple perspectives. From a neurocognitive standpoint, the complex motor sequencing, rapid decision-making, and inhibitory control required during martial arts practice may strengthen prefrontal cortex functioning and enhance top-down regulatory processes (Tang and Posner, 2009). The integration of mindfulness-like attention training within martial arts may facilitate metacognitive awareness and strengthen connections between attention networks and emotion regulation circuits (Hölzel et al., 2011). Additionally, the controlled exposure to physical and psychological stressors inherent in martial arts sparring and competition may enhance stress resilience through adaptive habituation mechanisms (Seery, 2011).

Despite growing interest in martial arts as a self-regulation intervention, several critical gaps remain in the literature. First, few randomized controlled trials have directly compared martial arts training to conventional physical exercise while controlling for key variables such as exercise intensity, duration, and frequency (Wang et al., 2025). Second, most studies have focused on single outcome domains rather than examining the comprehensive effects across emotional regulation, attentional control, and stress resilience simultaneously. Third, limited research has incorporated objective neurophysiological measures alongside self-report assessments to provide converging evidence for martial arts effects on self-regulatory processes.

### 1.1 Theoretical framework

The present study is grounded in an integrative theoretical framework drawing upon Self-Regulation Theory, Stress Inoculation Theory, and mindfulness-based models of attentional regulation. These complementary perspectives collectively explain how repeated engagement in cognitively demanding, emotionally challenging, and physiologically arousing contexts may strengthen regulatory capacity across psychological and autonomic domains. Martial arts training represents a multimodal behavioral context in which these regulatory systems are repeatedly engaged. First, structured motor sequencing and reaction-based drills require sustained attention and executive inhibition, aligning with neurocognitive models of top-down attentional control. Second, controlled sparring and performance-based challenges introduce graded stress exposure consistent with Stress Inoculation Theory, facilitating adaptive stress appraisal and physiological regulation. Third, breath regulation and mindful movement practices embedded in martial arts parallel mechanisms identified in mindfulness-based interventions, promoting interoceptive awareness and parasympathetic modulation. Accordingly, we conceptualize martial arts training as a structured self-regulation training environment in which cognitive control, emotional modulation, and stress adaptation mechanisms are simultaneously activated. This integrative framework guided the selection of primary outcomes (emotion regulation, attentional control, perceived stress) and secondary neurophysiological markers (electrodermal activity, executive inhibition).

The present study addressed these gaps through a randomized controlled trial comparing martial arts self-regulation training with conventional physical exercise and a passive control group. We employed a multimethod assessment approach incorporating validated psychometric instruments and objective measures of attentional performance, cognitive flexibility, and autonomic stress reactivity. Based on the theoretical framework and existing evidence, we hypothesized that martial arts training would produce superior improvements in emotion regulation, attentional control, and stress resilience compared to both conventional exercise and control conditions, reflecting the unique cognitive-emotional demands of martial arts practice.

## 2 Materials and methods

### 2.1 Participants of the study

A total of 66 healthy university-aged young adults (18–25 years), consistent with developmental definitions of emerging adulthood. National-level status was defined as athletes who had represented their state in officially recognized national championships within the past 3 years. Eligibility criteria included: (a) no prior formal training in martial arts, (b) absence of diagnosed psychological, neurological, or cardiovascular disorders, (c) no engagement in structured mindfulness or meditation practices during the previous 6 months, and (d) willingness to provide written informed consent. Participants with musculoskeletal injuries or medical conditions contraindicating physical activity were excluded. An *a priori* sample size estimation was performed using G*Power (version 3.1.9.7) based on a mixed-design analysis of variance (ANOVA) with three groups and two repeated measurements. The following parameters were applied: a medium effect size (*f* = 0.25), an alpha level of 0.05, statistical power of 0.80, three groups, two measurement occasions, an assumed correlation among repeated measures of 0.50, and a non-sphericity correction of *ε* = 1. The analysis indicated a minimum required total sample size of 66 participants (22 per group). To account for potential attrition and incomplete data, the recruitment target was increased by approximately 15–20%, resulting in an anticipated sample size of 75–80 participants (see Tables 1–5).

**Table 1:** Demographic characteristics of participants across study groups.

Variable	MA-SRT mean ± SD	CPEG mean ± SD	Control mean ± SD	*F*	*p*
Age (years)	21.4 ± 1.9	21.1 ± 2.1	21.3 ± 2.0	0.11	*0.90*
Height (cm)	170.8 ± 6.4	171.2 ± 6.7	170.5 ± 6.2	0.08	*0.92*
Body weight (kg)	66.9 ± 7.8	67.4 ± 8.1	66.7 ± 7.6	0.06	*0.94*
Body Mass Index (kg·m^−2^)	22.9 ± 2.3	23.0 ± 2.4	22.8 ± 2.2	0.09	*0.91*
Playing experience (years)	5.1 ± 1.8	5.3 ± 1.9	5.0 ± 1.7	0.13	*0.88*

Values are presented as mean ± standard deviation. No significant differences were observed between groups for any demographic variable (*p* > 0.05), indicating baseline equivalence.

**Table 2:** Martial arts self-regulation training group (MA-SRT).

Week	Training focus	Session structure (60 min)	Intensity	Density/psychological load
1	Familiarization & basic control	Basic stances, slow punches/kicks, breathing awareness, simple forms	Low	Low emotional arousal, high instructor guidance
2	Motor precision & attentional focus	Technique drills, slow kata/forms, breath-movement synchronization	Low–moderate	Increased attentional demand
3	Controlled movement sequencing	Complex forms, movement transitions, rhythm control	Moderate	Sustained attentional focus
4	Emotional awareness under movement	Forms + light partner drills (non-contact), breathing regulation	Moderate	Mild emotional arousal
5	Stress exposure & regulation	Controlled sparring drills (non-contact), decision-making tasks	Moderate–high	Emotional regulation under pressure
6	Cognitive-emotional integration	Faster forms, reaction-based drills, regulated breathing	High	High attentional & emotional load
7	Performance consistency	Continuous sequences, minimal instructor feedback	High	Self-regulated attention & emotion
8	Peak self-regulation	Complex drills, simulated performance stress, recovery breathing	High	Maximum cognitive-emotional control

The MA-SRT program was delivered three times per week for 60 min per session over 8 weeks. Training intensity progressed gradually from low to high and incorporated structured movement practice, controlled breathing, attentional focus, and regulated exposure to psychological stress. All sessions were supervised by a qualified martial arts instructor to ensure consistency and participant safety.

**Table 3:** Conventional physical exercise group (CPEG).

Week	Training focus	Session structure (60 min)	Intensity	Density
1	Familiarization	Brisk walking, light mobility, bodyweight exercises	Low	Low
2	Aerobic conditioning	Walking/jogging intervals, mobility drills	Low–moderate	Moderate
3	Cardiovascular load	Continuous jogging, basic resistance exercises	Moderate	Moderate
4	Muscular endurance	Circuit training (bodyweight), aerobic segments	Moderate	Moderate–high
5	Progressive overload	Increased repetitions and aerobic duration	Moderate–high	High
6	Combined aerobic-strength	Longer circuits, reduced rest intervals	High	High
7	Peak conditioning	Continuous aerobic work, full circuits	High	High
8	Maintenance	Stable high-intensity workload	High	High

The CPEG program was matched to the MA-SRT intervention in session duration, frequency, and overall exercise intensity. Training focused exclusively on aerobic and resistance exercises and did not include components related to attentional training, emotional regulation, or mindfulness. Sessions were supervised to ensure adherence and safety.

**Table 4:** Training intensity progression for intervention groups.

Week	MA-SRT group (martial arts)	RPE (Borg 6–20)	HR zone (%HRmax)	CPEG group (physical exercise)	RPE	HR zone
1	Basic techniques, slow forms	9-10	50–55%	Brisk walking, mobility	9-10	50%–55%
2	Technique drills + breathing	10-11	55–60%	Walking-jogging intervals	10-11	55%–60%
3	Continuous forms, sequencing	11-12	60–65%	Continuous jogging	11-12	60%–65%
4	Forms + light partner drills	12-13	65–70%	Aerobic + light resistance	12-13	65%–70%
5	Controlled sparring drills	13-14	70–75%	Circuit-based training	13-14	70%–75%
6	Reaction-based drills	14-15	75–80%	Reduced-rest circuits	14-15	75%–80%
7	Continuous performance sets	15-16	80–85%	High-load conditioning	15-16	80%–85%
8	Simulated competitive stress	15–17	80–88%	Peak conditioning	15–17	80%–88%

Training intensity for both intervention groups was monitored using the Borg Rating of Perceived Exertion (RPE; 6–20 scale) and estimated heart rate zones expressed as a percentage of age-predicted maximal heart rate (%HRmax). Intensity progression was matched between groups across the intervention period to control for exercise load, while the training content differed according to intervention type. All sessions were supervised to ensure adherence, safety, and consistency.

**Table 5:** Intra-class correlation coefficients (ICC) for outcome measures.

Outcome variable	ICC (95% CI)
Emotion regulation (ERQ)	0.88
Attention RT (CPT)	0.91
Perceived stress (PSS-10)	0.87
Psychological resilience (CD-RISC)	0.90
Electrodermal activity (EDA)	0.89
Stroop interference (ms)	0.92

ICC values were calculated using a two-way mixed-effects model with absolute agreement. Reliability interpretation followed conventional thresholds: <0.50 = poor, 0.50–0.75 = moderate, 0.75–0.90 = good, and >0.90 = excellent.

### 2.2 Study design

The present study employed a randomized controlled experimental design with three parallel groups and repeated measurements conducted before and after the intervention period. The experimental framework followed a 3 (Group: Martial Arts Training, Conventional Physical Exercise, Control) × 2 (Time: Pre-intervention, post-intervention) mixed factorial design. Participants were recruited using a purposive sampling technique, ensuring that all individuals met the predefined inclusion criteria relevant to psychological self-regulation research. Following baseline assessment, participants were randomly assigned to one of the three study groups using a computer-generated randomization sequence to minimize allocation bias and enhance internal validity.

### 2.3 Intervention procedures

#### 2.3.1 Martial arts self-regulation training group (MA-SRT)

Participants in the MA-SRT group underwent a structured martial arts training program (e.g., Karate or Taekwondo) specifically designed to enhance psychological self-regulation. The intervention lasted 8–12 weeks, with three sessions per week, each lasting approximately 60 min.

#### 2.3.2 Conventional physical exercise group (CPEG)

Participants in the CPEG group engaged in general physical exercise training, including aerobic activities (e.g., jogging, cycling) and basic resistance exercises. Session duration, frequency, and approximate intensity were matched to the martial arts group. No components related to attentional focus, emotional regulation, or mindfulness were included. To preserve the internal validity of the active control condition, instructors delivering the CPEG intervention were explicitly instructed to focus exclusively on physiological conditioning components (aerobic endurance and resistance exercises). The intervention protocol deliberately excluded structured attentional guidance, emotional regulation instruction, breath–movement synchronization, mindfulness cues, or reflective self-monitoring practices. Coaches were trained to avoid emphasizing psychological regulation strategies during sessions to minimize conceptual overlap with the mechanisms targeted in the MA-SRT. This procedural distinction was maintained throughout the intervention period to ensure interpretive clarity of between-group differences.

#### 2.3.3 Control group (CG)

Participants assigned to the control group did not receive any structured physical or psychological intervention during the eight-week study period. They were instructed to maintain their usual daily routines and to refrain from initiating any new exercise, martial arts practice, mindfulness activities, or psychological training programs throughout the intervention period. This group served as a passive control condition to allow comparison with the intervention groups and to account for natural variations over time.

### 2.4 Outcome measures

All outcome variables were assessed 1 week before (pre-test) and 1 week after (post-test) the intervention period by trained assessors blinded to group allocation. To ensure conceptual clarity and minimize outcome multiplicity, study variables were categorized as primary and secondary outcomes based on their direct relevance to the study aims and title. A combination of validated psychometric instruments and objective neurophysiological measures was employed.

#### 2.4.1 Primary outcome measures

##### 2.4.1.1 Emotion regulation

Emotion regulation was assessed using the Emotion Regulation Questionnaire (ERQ), which evaluates individual differences in cognitive reappraisal and expressive suppression strategies. The ERQ has demonstrated good reliability and validity in adult populations and is widely used in self-regulation research (Aune et al., 2025).

##### 2.4.1.2 Attentional control

Attentional control was assessed with a computerized Continuous Performance Task (CPT) implemented on the PsyToolkit platform (Stoet, 2010). Primary outcome measures were reaction time and error rates, which served as objective indices of sustained attention and inhibitory control (Riccio et al., 2001).

##### 2.4.1.3 Perceived stress

Perceived stress was assessed using the Perceived Stress Scale (PSS-10), which measures the degree to which individuals appraise life situations as stressful (Cohen et al., 1983). The PSS-10 is a validated and commonly used instrument in psychological and stress-related research (Saha et al., 2025).

#### 2.4.2 Secondary outcome measures

##### 2.4.2.1 Psychological resilience

Psychological resilience was evaluated using the Connor-Davidson Resilience Scale (CD-RISC-25) (Connor and Davidson, 2003), which assesses adaptability, coping capacity, and psychological strength in the face of stress (Campbell-Sills and Stein, 2007).

##### 2.4.2.2 Emotional arousal and stress reactivity

Emotional arousal and stress reactivity were objectively assessed through electrodermal activity (EDA) using the Empatica E4 wearable sensor (Garbarino et al., 2014). Skin conductance level and response frequency were extracted as indicators of sympathetic nervous system activation (Boucsein, 2012; Critchley, 2002). EDA was included to complement self-reported stress measures with objective physiological data.

##### 2.4.2.3 Cognitive flexibility and executive control

Cognitive flexibility and executive inhibitory control were assessed using a computerized Stroop Color-Word Task administered via Inquisit Lab software. Reaction time interference scores and accuracy rates were used as indices of executive control processes relevant to psychological self-regulation (MacLeod, 1991; Scarpina and Tagini, 2017).

### 2.5 Ethical considerations

The study protocol was reviewed and approved by the Institutional Ethics Committee of Guangdong University of Petrochemical Technology, China (Approval No.: IEC/GUPT/SPE718). All procedures were conducted in accordance with the ethical principles outlined in the Declaration of Helsinki and its later amendments. Prior to participation, all participants received detailed information regarding the study objectives, procedures, potential risks, and benefits, and provided written informed consent. Participants were informed of their right to withdraw from the study at any time without any penalty or consequences. Confidentiality and anonymity were ensured through the use of coded identifiers, and all data were securely stored and accessed only by the research team.

### 2.6 Statistical analysis

Data were analyzed using SPSS (version 23). Descriptive statistics (mean ± standard deviation) were calculated for all variables. The normality of data distribution was assessed using the Shapiro–Wilk test. To control for Type, I error arising from multiple outcome measures, a hierarchical analysis strategy was adopted. Primary outcome variables were analyzed first, followed by secondary outcomes only if significant group × time interactions were observed in the primary analyses. For primary outcomes, a mixed-model repeated-measures ANOVA was conducted with group (martial arts training, conventional exercise, control) as the between-subjects factor and time (pre, post) as the within-subjects factor. Where baseline differences were present, ANCOVA was performed with baseline values entered as covariates. For secondary outcomes, the same analytical approach was applied; however, Bonferroni-adjusted *post hoc* comparisons were used to further control the family-wise error rate. Effect sizes were reported using partial eta squared (*η*^2^*_p_*) for ANOVA effects and Cohen’s *d* for pairwise comparisons. Statistical significance was set at *p* < 0.05 for primary outcomes. For secondary outcomes, adjusted significance thresholds were applied where appropriate. This analytical approach ensured a balance between statistical rigor and interpretability while minimizing the risk of false-positive findings. EDA was included as an objective indicator of sympathetic stress reactivity to complement self-reported stress measures. The Stroop task was used to assess executive inhibitory control, a core mechanism underlying psychological self-regulation.

## 3 Results

Table 6 presents the baseline comparison of psychological and neurophysiological variables across the MA-SRT, CPEG, and control group. One-way analyses of variance revealed no significant differences between groups at baseline for emotion regulation (ERQ), attentional reaction time, perceived stress, psychological resilience, EDA, or Stroop interference scores (all *p* > 0.05). These findings indicate successful randomization and baseline equivalence across all outcome measures prior to the intervention.

**Table 6:** Baseline comparison of psychological and neurophysiological variables among the martial arts self-regulation training, conventional physical exercise, and control groups.

Variable	MA-SRT (*n* = 22) mean ± SD	CPEG (*n* = 22) mean ± SD	Control (*n* = 22) mean ± SD	*F*	*p*
Emotion regulation (ERQ)	3.81 ± 0.49	3.79 ± 0.52	3.77 ± 0.51	0.08	*0.92*
Attention RT (ms)	421.6 ± 34.8	418.9 ± 36.1	419.8 ± 35.4	0.06	*0.94*
Perceived stress (PSS)	21.3 ± 4.1	21.0 ± 3.9	21.1 ± 4.0	0.04	*0.96*
Psychological resilience (CD-RISC)	64.8 ± 6.2	65.1 ± 6.4	64.9 ± 6.1	0.02	*0.98*
Electrodermal activity (μS)	4.82 ± 0.71	4.79 ± 0.69	4.81 ± 0.72	0.01	*0.99*
Stroop interference (ms)	121.4 ± 19.6	119.8 ± 20.4	120.7 ± 19.9	0.05	*0.95*

Table 7 summarizes the pre–post changes in primary psychological self-regulation outcomes across the MA-SRT, CPEG, and Control Group. Mixed-model analyses revealed significant group × time effects for emotion regulation, attentional reaction time, and perceived stress (all *p* < 0.001). The MA-SRT group demonstrated the largest improvements, characterized by increased emotion regulation scores, substantial reductions in attention reaction time, and marked decreases in perceived stress, whereas the CPEG group showed moderate improvements and the control group exhibited minimal change. The magnitude of these effects was large, as indicated by partial eta squared values ranging from 0.49 to 0.75, reflecting strong intervention-related effects on primary self-regulation outcomes.

**Table 7:** Group differences in pre-post changes for primary outcome variables across the intervention period.

Variable	MA-SRT Δ (post-pre)	CPEG Δ (post-pre)	Control Δ (post-pre)	*F*	*p*	*η* ^2^ *_p_*
Emotion regulation (ERQ)	+0.92	+0.36	+0.11	30.70	*<0.001*	0.49
Attention RT (ms)	−59.87	−30.03	−5.02	93565.52	*<0.001*	0.75
Perceived stress (PSS)	−6.09	−2.93	−0.47	1871.56	*<0.001*	0.63

Δ indicates mean change scores (post-pre). Effect sizes are reported as partial eta squared (*η*^2^*_p_*). Values of 0.01, 0.06, and 0.14 represent small, medium, and large effects, respectively.

Table 8 presents the pre-post changes in secondary psychological and neurophysiological outcomes across the MA-SRT, CPEG, and control group. Significant group × time effects were observed for psychological resilience, EDA, and Stroop interference (all *p* < 0.001). Participants in the MA-SRT group exhibited the greatest improvements, reflected by substantial increases in resilience and pronounced reductions in sympathetic arousal and executive interference, whereas the CPEG group demonstrated moderate changes and the control group showed minimal variation. The effect sizes were large, with partial eta squared values ranging from 0.53 to 0.71, indicating robust intervention effects on secondary self-regulation-related outcomes.

**Table 8:** Group differences in pre-post changes for secondary outcome measures.

Variable	MA-SRT Δ (post-pre)	CPEG Δ (post-pre)	Control Δ (post-pre)	*F*	*p*	*η* ^2^ *_p_*
Psychological resilience (CD-RISC)	+10.01	+3.98	+0.89	3061.71	*<0.001*	0.69
Electrodermal activity (μS)	−1.13	−0.54	−0.12	35.50	*<0.001*	0.53
Stroop interference (ms)	−35.04	−14.94	−2.97	39843.55	*<0.001*	0.71

Δ indicates mean change scores (post-pre). Effect sizes are reported as partial eta squared (*η*^2^*_p_*). Values of 0.01, 0.06, and 0.14 represent small, medium, and large effects, respectively.

Table 9 reports the Bonferroni-adjusted *post hoc* pairwise comparisons for primary psychological self-regulation outcomes. Significant differences were observed between the MA-SRT group and both the CPEG and control groups for emotion regulation, attentional reaction time, and perceived stress (all *p* < 0.001), with large effect sizes (Cohen’s *d* = 0.84–1.34). Comparisons between the CPEG and control groups also reached statistical significance across all primary outcomes (*p* ≤ 0.032), although the associated effect sizes were smaller. Confidence intervals indicated greater improvements in emotion regulation and larger reductions in attentional reaction time and perceived stress in the MA-SRT group relative to the comparison groups.

**Table 9:** *Post Hoc* pairwise comparisons between groups for primary outcome variables following the intervention.

Outcome variable	Group comparison	95% confidence interval	*p* (Bonferroni)	Cohen’s *d*
Emotion regulation (ERQ)	MA-SRT vs. CPEG	[0.31, 0.81]	*<0.001*	0.84
	MA-SRT vs. control	[0.54, 1.08]	*<0.001*	1.21
	CPEG vs. control	[0.02, 0.48]	*0.032*	0.42
Attention RT (ms)	MA-SRT vs. CPEG	[−38.71, −20.97]	*<0.001*	0.91
	MA-SRT vs. control	[−64.29, −45.41]	*<0.001*	1.34
	CPEG vs. control	[−33.79, −16.23]	*<0.001*	0.72
Perceived stress (PSS)	MA-SRT vs. CPEG	[−4.44, −1.88]	*<0.001*	0.88
	MA-SRT vs. control	[−7.02, −4.22]	*<0.001*	1.29
	CPEG vs. control	[−3.69, −1.23]	*0.001*	0.61

Bonferroni-adjusted *p*-values are reported for all pairwise comparisons. Effect sizes are expressed as Cohen’s *d*. Positive confidence intervals for emotion regulation indicate greater improvement in the first-listed group, whereas negative confidence intervals for attention reaction time and perceived stress indicate greater reductions (i.e., improvement) in the first-listed group.

Table 10 reports the Bonferroni-adjusted *post hoc* pairwise comparisons for secondary psychological and neurophysiological outcomes. Significant differences were observed between the MA-SRT group and both the CPEG and control groups for psychological resilience, EDA, and Stroop interference (all *p* < 0.001), with large effect sizes (Cohen’s *d* = 0.83–1.36). Comparisons between the CPEG and control groups were also statistically significant across all secondary outcomes (*p* ≤ 0.004), although the associated effect sizes were comparatively smaller. The confidence intervals indicated greater increases in psychological resilience and larger reductions in sympathetic arousal and executive interference in the MA-SRT group relative to the comparison groups.

**Table 10:** *Post Hoc* group comparisons for secondary outcome variables following the intervention.

Outcome variable	Group comparison	95% confidence interval	*p*	Cohen’s *d*
Psychological resilience (CD-RISC)	MA-SRT vs. CPEG	[3.95, 8.11]	*<0.001*	0.97
	MA-SRT vs. control	[6.90, 11.34]	*<0.001*	1.36
	CPEG vs. control	[1.07, 5.11]	*0.004*	0.52
Electrodermal activity (μS)	MA-SRT vs. CPEG	[−0.88, −0.30]	*<0.001*	0.83
	MA-SRT vs. control	[−1.32, −0.70]	*<0.001*	1.25
	CPEG vs. control	[−0.69, −0.15]	*0.003*	0.59
Stroop interference (ms)	MA-SRT vs. CPEG	[−27.52, −12.68]	*<0.001*	0.94
	MA-SRT vs. control	[−40.05, −24.09]	*<0.001*	1.31
	CPEG vs. control	[−19.25, −4.69]	*0.002*	0.56

Bonferroni-adjusted *p*-values are reported for all pairwise comparisons. Effect sizes are expressed as Cohen’s *d*. Positive confidence intervals for psychological resilience indicate greater improvement in the first-listed group, whereas negative confidence intervals for electro-dermal activity and Stroop interference indicate greater reductions (i.e., improvement) in the first-listed group.

## 4 Discussion

### 4.1 Interpretation of finding

The present study examined the effects of martial arts self-regulation training on emotion regulation, attentional control, and stress resilience in healthy young adults. The term “young adults” in this study refers specifically to university-aged individuals between 18 and 25 years, and findings should be interpreted within this developmental context. The findings demonstrate that an 8-week structured martial arts intervention produced significantly greater improvements in psychological self-regulation compared to conventional physical exercise and passive control conditions. Participants in the MA-SRT group exhibited enhanced emotion regulation, faster attentional processing, reduced perceived stress, increased psychological resilience, decreased sympathetic arousal, and improved executive inhibitory control. These results provide empirical support for the role of martial arts training as an effective psychological self-regulation intervention.

The most robust finding of this study was the substantial improvement in emotion regulation among participants who completed the martial arts training program. The MA-SRT group demonstrated a mean increase of 0.92 points on the ERQ, significantly outperforming both the conventional exercise group (Δ = +0.36) and the control group (Δ = +0.11), with a large effect size (*η*^2^*_p_* = 0.49). This finding aligns with previous research indicating that martial arts training enhances emotional regulation capacities through repeated exposure to controlled stress and the cultivation of mindful awareness during movement (Origua Rios et al., 2018). Lakes and Hoyt (2004) similarly reported improvements in self-regulation among children following a Taekwondo intervention, suggesting that the integration of physical discipline with cognitive and emotional control may facilitate adaptive regulatory strategies across developmental periods (Lakes and Hoyt, 2004). The emphasis on breath control, focused attention, and deliberate movement sequencing inherent in martial arts practice appears to create a training environment conducive to the development of cognitive reappraisal strategies, which are central to effective emotion regulation (Gross, 2015).

Attentional control, as measured by reaction time on the Continuous Performance Task, showed the largest intervention effect observed in this study (*η*^2^*_p_* = 0.75). Participants in the MA-SRT group exhibited a mean reduction of 59.87 ms in reaction time, substantially exceeding the improvements observed in the CPEG (Δ = −30.03 ms) and control groups (Δ = −5.02 ms). These findings are consistent with research demonstrating that martial arts training enhances sustained attention and response inhibition (Lakes and Hoyt, 2004; Diamond and Ling, 2016; Goethel et al., 2023). The repetitive practice of kata or forms, which requires precise timing, sequencing, and sustained concentration, may strengthen neural networks associated with attentional control and executive function (Tang and Posner, 2009). Furthermore, the progressive introduction of controlled sparring and decision-making tasks under time pressure likely enhanced participants’ ability to maintain focus and rapidly process relevant stimuli, skills that are reflected in reduced reaction times.

Perceived stress, as assessed by the PSS-10, decreased significantly in the MA-SRT group (Δ = −6.09) compared to the CPEG (Δ = −2.93) and control groups (Δ = −0.47), with a large effect size (*η*^2^*_p_* = 0.63). This result is in accordance with prior studies showing that martial arts practice reduces perceived stress and anxiety (Woodward, 2009; Zivin et al., 2001). The structured nature of martial arts training, which combines physical exertion with mental discipline and breath regulation, may activate parasympathetic recovery mechanisms and promote psychological detachment from daily stressors (Bu et al., 2010). Additionally, the mastery experiences gained through progressive skill acquisition in martial arts may enhance self-efficacy and perceived control, both of which are protective factors against stress (Bandura, 1997). The integration of mindfulness-like components within martial arts training may further contribute to stress reduction by fostering present-moment awareness and nonjudgmental acceptance of internal experiences (Moore and Malinowski, 2009).

Psychological resilience, as measured by the CD-RISC-25, improved markedly in the MA-SRT group (Δ = +10.01) relative to the CPEG (Δ = +3.98) and control groups (Δ = +0.89), with a large effect size (*η*^2^*_p_* = 0.69). Resilience development in martial arts may be facilitated by exposure to controlled physical and psychological challenges, which provide opportunities for participants to practice tolerance of discomfort, regulate emotional responses under pressure, and develop confidence in their ability to overcome adversity (Connor and Davidson, 2003). The philosophical and ethical components often embedded in martial arts training, such as perseverance, respect, and humility, may also contribute to resilience by fostering a growth-oriented mindset and a sense of purpose (Fuller, 1988).

EDA, an objective marker of sympathetic nervous system activation, decreased significantly in the MA-SRT group (Δ = −1.13 μS) compared to the CPEG (Δ = −0.54 μS) and control groups (Δ = −0.12 μS), with a moderate-to-large effect size (*η*^2^*_p_* = 0.53). This physiological finding complements the self-reported stress data and suggests that martial arts training may modulate autonomic reactivity to stressors (Critchley, 2002). The integration of controlled breathing techniques and mindful movement in martial arts practice has been shown to enhance vagal tone and reduce sympathetic dominance, thereby promoting physiological relaxation and stress resilience (Ditto et al., 2006; Zaccaro et al., 2018). These neurophysiological adaptations may reflect improved regulatory capacity at both psychological and biological levels.

Executive inhibitory control, assessed via Stroop interference scores, improved substantially in the MA-SRT group (Δ = −35.04 ms) relative to the CPEG (Δ = −14.94 ms) and control groups (Δ = −2.97 ms), with a large effect size (*η*^2^*_p_* = 0.71). This result is consistent with evidence indicating that martial arts training enhances executive function and cognitive control (Chang et al., 2013; Gu et al., 2019). The Stroop task requires participants to inhibit prepotent responses and resolve cognitive conflict, processes that are central to self-regulation (MacLeod, 1991). The martial arts training protocol employed in this study emphasized rapid decision-making, attentional switching, and inhibitory control during sparring and reaction-based drills, which may have strengthened prefrontal cortical networks involved in executive function (Diamond, 2013). These findings suggest that martial arts training may serve as a potent intervention for enhancing top-down cognitive control mechanisms.

While the CPEG demonstrated moderate improvements across most outcome measures, the magnitude of change was consistently smaller than that observed in the MA-SRT group. This pattern of results suggests that the unique combination of physical, cognitive, and emotional components inherent in martial arts training confers additional psychological benefits beyond those associated with general aerobic and resistance exercise. Previous research has similarly found that martial arts interventions outperform conventional exercise in promoting self-regulation and executive function (Lakes and Hoyt, 2004; Vertonghen and Theeboom, 2010). The integration of mindfulness, controlled breathing, structured movement sequences, and progressive exposure to controlled stress in martial arts training may activate distinct psychological and neurobiological pathways that are not fully engaged by conventional exercise alone (Gothe and McAuley, 2015).

Nevertheless, the CPEG group did show statistically significant improvements compared to the control group across all primary and secondary outcomes, indicating that conventional physical exercise retains value as a psychological intervention. These findings are consistent with a substantial body of evidence demonstrating that regular physical activity enhances mood, reduces stress, and improves cognitive function (Erickson et al., 2011; Mandolesi et al., 2018). The cardiovascular and neurobiological adaptations associated with aerobic exercise, including increased hippocampal neurogenesis and elevated brain-derived neurotrophic factor (BDNF) levels, may partially account for these benefits (Cotman et al., 2007).

### 4.2 Theoretical implications

The superior psychological outcomes observed in the MA-SRT group may be explained by several interrelated mechanisms. First, martial arts training integrates mindfulness and focused attention, which have been shown to enhance self-regulation and reduce stress reactivity (Kabat-Zinn, 2003; Tang et al., 2015). The deliberate coordination of breath and movement in martial arts practice closely resembles mindfulness meditation, promoting present-moment awareness and nonjudgmental observation of internal states (Moore and Malinowski, 2009). Second, the progressive exposure to controlled physical and psychological challenges in martial arts training may function as a form of stress inoculation, enhancing participants’ ability to remain calm and focused under pressure (Meichenbaum, 1985). Third, the mastery experiences and skill progression inherent in martial arts practice may strengthen self-efficacy and intrinsic motivation, both of which are associated with improved psychological well-being (Deci and Ryan, 2000; Zimmerman, 2000). Fourth, the structured and ritualized nature of martial arts training may provide a sense of order, discipline, and purpose, which can buffer against stress and promote psychological resilience (Vertonghen and Theeboom, 2010).

From a neurobiological perspective, martial arts training may enhance self-regulation by modulating activity in brain regions associated with executive function, emotion regulation, and interoceptive awareness, including the prefrontal cortex, anterior cingulate cortex, and insula (Tang et al., 2012; Craig, 2009). Neuroimaging studies have demonstrated that mindfulness-based interventions and martial arts practice are associated with structural and functional changes in these regions (Hölzel et al., 2011; Raine et al., 2018). Additionally, the integration of aerobic exercise with cognitive training in martial arts may produce synergistic effects on neuroplasticity and cognitive function (Fabel et al., 2009).

Taken together, the present findings suggest that these theoretical mechanisms operate in a synergistic rather than independent manner within the context of martial arts training. The simultaneous improvements observed across executive inhibitory control, attentional performance, perceived stress, and autonomic regulation indicate coordinated engagement of cognitive and physiological regulatory systems. While neurocognitive models explain enhancements in top-down executive processes, Stress Inoculation Theory accounts for adaptive modulation of stress reactivity, and mindfulness-based perspectives elucidate the role of breath regulation and metacognitive awareness. Importantly, the convergence of these outcomes within a single intervention framework extends prior literature by empirically demonstrating that martial arts training may function as an integrated self-regulation training environment in which executive control, stress adaptation, and mindful attentional engagement interact to produce comprehensive psychological benefits. Thus, the theoretical contribution of the present study lies in highlighting the synergistic integration of these regulatory mechanisms rather than attributing the effects to a single explanatory model.

### 4.3 Practical and educational implications

The findings of this study have several practical implications. First, martial arts training may be a viable and effective intervention for enhancing psychological self-regulation in young adults, with potential applications in educational, clinical, and community settings. Schools and universities could incorporate martial arts programs to promote students’ emotional and cognitive development, particularly in populations at risk for stress-related difficulties (Theeboom et al., 2009). Second, clinicians and mental health professionals may consider recommending martial arts training as a complementary approach to traditional psychotherapy for individuals seeking to improve emotion regulation, reduce stress, and build resilience (Castonguay et al., 2012). Third, organizations and workplaces may benefit from offering martial arts-based wellness programs to enhance employees’ stress management skills and overall well-being (Richardson and Rothstein, 2008).

### 4.4 Clinical and community applications

Beyond higher education contexts, martial arts-based self-regulation programs may hold promise for other populations characterized by elevated stress or regulatory demands. For example, competitive athletes, high-performance professionals, and individuals working in high-pressure occupations may benefit from structured training that integrates attentional control, emotional regulation, and stress exposure. Additionally, individuals experiencing stress-related symptoms or subclinical emotional dysregulation may potentially benefit from such multimodal interventions. However, extrapolation beyond the present university-aged sample requires empirical validation. The psychological, physiological, and contextual characteristics of different populations may influence intervention effectiveness. Future randomized controlled trials should examine adaptation of martial arts-based self-regulation protocols for clinical populations, older adults, occupational groups, and sport-specific contexts to determine feasibility, safety, and effectiveness.

### 4.5 Limitations of the study

Several limitations should be acknowledged. First, the sample consisted exclusively of healthy university-aged young adults (18–25 years), which limits generalizability to adolescents, older adults, clinical populations, or individuals with lower baseline psychological functioning. The relative homogeneity and developmental plasticity of this population may also have influenced the magnitude of observed effect sizes. Replication in more diverse and heterogeneous samples is necessary to determine the stability and generalizability of the findings. Second, although an active control group was employed, the study did not include a condition that matched the social interaction and instructional structure of martial arts training. Future research incorporating attention-control designs would further clarify specific versus nonspecific intervention effects. Third, the intervention duration was limited to 8 weeks. Longitudinal research with follow-up assessments is required to determine the durability of psychological and physiological adaptations. Finally, potential mediating mechanisms (e.g., changes in mindfulness, self-efficacy, or autonomic regulation patterns) were not directly tested. Future studies employing mediation analyses could clarify the pathways through which martial arts training enhances self-regulation (Duncan et al., 2010). Future studies should incorporate attention-control conditions to disentangle specific from nonspecific intervention effects. Fourth, the study did not assess potential mediating mechanisms, such as changes in mindfulness, self-efficacy, or autonomic nervous system regulation, which could provide deeper insights into how martial arts training enhances self-regulation. Finally, the study was conducted in a specific cultural context, and cultural factors may influence both the practice of martial arts and the interpretation of psychological constructs such as emotion regulation and resilience (Tsai et al., 2006).

### 4.6 Future research directions

Several avenues for future research emerge from this study. First, neuroimaging studies could elucidate the neural mechanisms underlying the psychological benefits of martial arts training by examining changes in brain structure and function associated with emotion regulation, attention, and executive control (Tang et al., 2012). Second, dose–response studies could investigate the optimal frequency, intensity, and duration of martial arts training needed to produce meaningful psychological improvements. Third, comparative effectiveness research could examine whether different styles of martial arts (e.g., karate, taekwondo, judo, aikido) produce differential effects on psychological outcomes. Fourth, mediation and moderation analyses could identify the psychological, behavioral, and physiological pathways through which martial arts training enhances self-regulation and determine which individuals are most likely to benefit from such interventions. Finally, implementation research could explore the feasibility, acceptability, and scalability of martial arts-based interventions in real-world settings, including schools, mental health clinics, and community centers.

## 5 Conclusion

This randomized controlled trial provides evidence that structured martial arts training enhances multiple dimensions of psychological self-regulation, including emotion regulation, attentional control, stress resilience, executive function, and autonomic modulation. Compared with intensity-matched conventional physical exercise, martial arts training produced larger and more consistent improvements across psychological and physiological domains. The findings support an integrative model in which cognitive control, stress adaptation, and mindful attentional engagement interact to strengthen regulatory capacity. However, these conclusions are limited to healthy university-aged young adults, and generalization to younger, older, clinical, or more heterogeneous populations requires further empirical investigation. Continued research across diverse populations and contexts will help clarify the broader applicability and long-term impact of martial arts-based self-regulation interventions.

## Data Availability

The data supporting the findings of this study are available from the corresponding author upon reasonable request. Due to ethical considerations and the need to protect participant privacy, the datasets are not publicly deposited in a repository. De-identified data may be shared for academic and research purposes following approval from the Institutional Review Board of Guangdong University of Petrochemical Technology, China, and in compliance with applicable data protection regulations.
